# Plasma longitudinal metabolic changes with acute maximal aerobic exercise and one-hour recovery

**DOI:** 10.3389/fmolb.2025.1613238

**Published:** 2025-07-11

**Authors:** William A. Fountain, Stefano Donega, Nirad Banskota, Ruin Moaddel, Mona Patel, Linda Zukley, Sarah Church, Katie Bustin, Josephine M. Egan, Jeremy Walston, Luigi Ferrucci

**Affiliations:** ^1^ Division of Geriatric Medicine and Gerontology, Johns Hopkins University School of Medicine, Baltimore, MD, United States; ^2^ Longitudinal Study Section (LSS), Intramural Research Program (IRP), National Institute on Aging (NIH), Baltimore, MD, United States; ^3^ Computational Biology and Genomics Core (CBGC), Intramural Research Program (IRP), National Institute on Aging (NIH), Baltimore, MD, United States; ^4^ Laboratory of Clinical Investigation (LCI), Intramural Research Program (IRP), National Institute on Aging (NIH), Baltimore, MD, United States; ^5^ Clinical Research Core (CRC), Harbor Hospital, National Institute on Aging (NIH), Baltimore, MD, United States

**Keywords:** exercise, metabolomics, fatigability, fitness, energy, physiology, longitudinal, recovery

## Abstract

**Introduction:**

Total body metabolism continuously adapts to match energy supply with demand. During exercise metabolic alterations occur because skeletal muscles require a continuous supply of newly generated ATP to match the demand of the intensity of the exercise, and products of muscle metabolism must be eliminated. The metabolic and energetic flexibility greatly impact maximum physical fitness and exercise duration, as well as the speed of elimination of metabolism end-products. However, so far, the temporal profiling of metabolomic changes in response to exercise of persons with different fitness levels remains relatively unexplored. This study examined metabolic changes during each person’s peak aerobic exercise and one-hour post-exercise recovery in 29 Baltimore Longitudinal Study of Aging (BLSA) participants.

**Methods:**

Blood samples were collected at baseline, and at 3-min intervals during both incremental exercise on a treadmill until exhaustion and during recovery. Participants were classified based on the stage when they reached exhaustion as low fitness (LF, completing up to 3 treadmill incremental stages) or high fitness (HF, completing up to 7 incremental stages). The time course of exercise-associated changes in the circulating metabolome were mapped and unique metabolomic trajectories were identified with likelihood-ratio testing and hierarchical clustering.

**Results:**

The HF group had rapid clearance of bile and amino acids at exercise onset, along with effective clearance of triacylglycerols and glycerophospholipids during recovery. In contrast, the LF group had much reduced clearance of these metabolites and had persistent elevation of triacylglycerols and glycerophospholipids.

**Discussion:**

These findings highlight differences in bile acid clearance and purine metabolism in people of differing fitness levels and provide novel insights into the role of metabolic adaptive responses to aerobic exercise assessed through circulating metabolomic measures.

## 1 Introduction

Changes in energy metabolism continuously match fluctuations in energetic demand that occur in response to exogenous stressors. Acute metabolic responses are essential components of the downstream activation of transcriptional, inflammatory, and tissue remodeling mechanisms (Egan and Sharples, 2023). Moreover, the ability to return to, and maintain, a stable homeostatic baseline (i.e., recover) following the stressor is an important marker of resilience (Egan and Sharples, 2023; Egan et al., 2013). Indeed, the dynamic metabolic response profile to a stressor is often used to understand resilience capacity in epidemiological studies and clinical practice. For example, the cardiovascular and metabolic response to different levels of physical activity is traditionally used as a measure of physical fitness and coronary reserve.

Metabolic response to exercise and recovery patterns after exercise may provide clues about an individuals’ responses to various physical stressors (Walston et al., 2023; Lei et al., 2022; Ferrucci et al., 2020). For example, mapping trajectories of circulating metabolic responses to exercise can provide unique insight into mechanisms of differential fitness level across individuals.

Temporal profiling of metabolic responses to acute physical stress has been integral to the field of exercise physiology for decades (Costill, 1970; Craig et al., 1981; Romijn et al., 1993; Romijn et al., 1995; Bangsbo et al., 2002; Lavin et al., 2022). Such studies have laid the foundation for our ability to establish the relationships between physical activity levels, cardiovascular fitness, inflammation, metabolic health, chronic disease, mortality, and healthy aging (Steensberg et al., 2000; Gries et al., 2018; Perkins et al., 2020; Lavin et al., 2020; Chambers et al., 1985; Imboden et al., 2018; Imboden et al., 2019; Kaminsky et al., 2018). However, research in this field has not yet fully benefited from the new high-throughput technologies that can measure different biomarkers in biological fluids and analyze them with new powerful bioinformatic methods. Metabolomics has emerged as a powerful tool for investigating rapid physiological changes, offering significant advantages over traditional transcriptomic approaches. Metabolomics provides real-time insights into metabolic processes, including those influenced by post-transcriptional and post-translational modifications. The study of metabolites is particularly well-suited for detecting swift responses to environmental stimuli and systemic changes that may not be captured by transcriptomic analysis alone (Han et al., 2024; Wu et al., 2024). Thus, measuring a comprehensive profiling of the metabolic responses to exercise provides a unique opportunity to correlate these metabolic trajectories with level of physical fitness.

This study presents an innovative workflow designed to delineate distinct metabolic response patterns in individuals with variable cardiorespiratory fitness, which may provide new knowledge on the mechanism that drive differential risk for adverse health outcomes. Current research into exercise metabolomics focuses predominantly on steady-state exercise, with little insight toward the dynamic metabolic processes occurring during standard graded exercise testing (Jaguri et al., 2023). Our approach uses advanced high-throughput metabolomics and bioinformatics to generate a comprehensive and systemic profile of metabolic responses before, during, and after non-steady state aerobic exercise, building upon previous research (Contrepois et al., 2020; Parstorfer et al., 2025). This methodology captures rapid physiological changes at short intervals over a 2-h period, yielding novel insights into the metabolic responsiveness and physical fitness of middle-aged and older adults.

The overall aim of this study is to identify critical changes of metabolic pathways that could be considered as targets for interventions aimed at preventing the decline of exercise capacity that occur with aging and other condition and to monitor the effectiveness of such interventions.

## 2 Materials and methods

### 2.1 BLSA participants

Study participants belong to the Baltimore Longitudinal Study on Aging (BLSA) which has been in operation since 1958, standing out as one of the United States' longest-running investigations into healthy aging (Ferrucci, 2008). The BLSA employs a comprehensive approach, regularly assessing community-dwelling volunteers through a variety of clinical examinations, advanced imaging techniques, and extensive laboratory testing. It does not enroll participants at their first visit who have hip or knee joint replacement; severe knee osteoarthritis; history of stroke or Parkinson’s disease; or inability to walk without using a weight bearing assistive device. However, participants remain enrolled in the study as they develop chronic medical conditions, as well as physical and cognitive impairment over time. The BLSA protocol is approved by the Institutional Review Board of the Intramural Research Program of National Institutes of Health (IRB#03-AG-0325). All participants provided written informed consent.

### 2.2 Maximal aerobic exercise test

**FIGURE 1:**
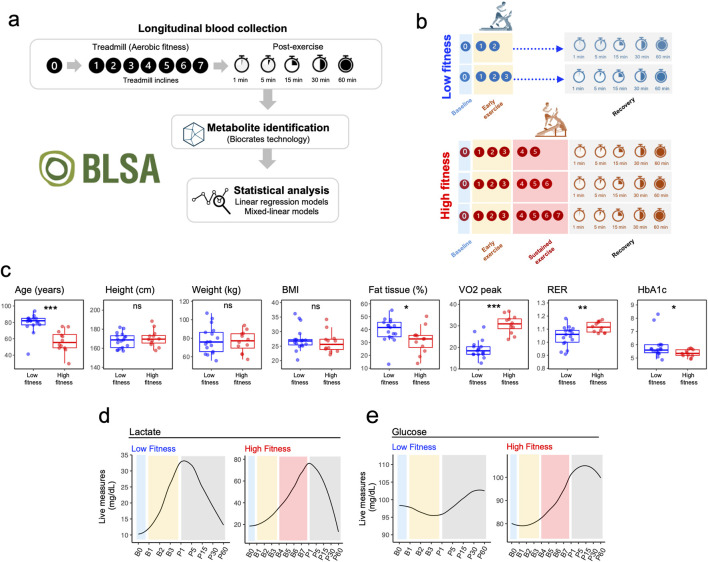
Experimental Design and Fitness Group Characteristic. **(a)** Schematic representation of the experimental protocol illustrating metabolomic sampling timepoints at baseline (rest, B0), during incremental exercise stages (B1-B7), and throughout the one-hour recovery period (P1-P60). **(b)** Fitness group stratification based on time to exhaustion during graded treadmill exercise. **(c)** Comparison of key physiological parameters between Low and High fitness groups, including Age, Height, Weight, BMI, Body Fat Percentage, VO_2_ peak, Respiratory Exchange Ratio, and Glucose levels. P-values from t-tests are indicated for each parameter. **(d)** Real-time lactic acid measurements (mg/dL) in Low (n = 17) and High (n = 12) fitness groups during exercise and recovery. **(e)** Real-time glucose measurements (mg/dL) in Low (n = 17) and High (n = 12) fitness groups during exercise and recovery.

Participant preparation involved placing 10 electrodes in standard stress EKG positions. A VO_2_ mask was fitted over the mouth and nose, and an O2 saturation monitor was placed on the earlobe. A blood pressure (BP) cuff was secured on the upper arm, and baseline EKG and BP were recorded prior to starting the test. The steady state protocol began with participants walking for 5 min at a speed of 1.5 mph. BP measurements were taken at 2 and 4 min during initial walking. Rate of perceived exertion (RPE) was recorded at the end of each testing intervals. The maximal treadmill protocol was designed with a variable duration and was contingent upon participants reaching volitional fatigue. For male participants, the test began at a speed of 3.5 mph with a 0% incline. After 45 s, the incline was increased to 3%, followed by increments of 3% every 3 min. For female participants, the protocol started at a speed of 3.0 mph with a 0% incline. Similar to the male protocol, the incline was increased to 3% after 45 s and followed a comparable pattern, ultimately reaching a maximum incline of 21% at the 18-min mark. During the exercise phase, BP measurements were taken every 2 min. The test continued until participants reported reaching maximum fatigue, unless contraindicated due to EKG changes, arrythmias or chest pain. In addition, the speed of the treadmill was adjusted to accommodate each participant’s physical ability, with the goal of achieving maximum heart rate. Following test termination, participants were asked to sit for the recovery phase. Oxygen consumption and BP were recorded during the 60 min of recovery time, and RPE was recorded at the end of recovery (Figure 1A).

### 2.3 Blood collection

A venous line was established for blood collection. The treadmill protocol continued seamlessly during blood draws, with participants maintaining their assigned incline and speed. To facilitate blood collection during active treadmill testing, participants were instructed to rest their arm on the handrail. Bloods were collected at predetermined time points: baseline, within the first minute and every 3 min of each treadmill grade or speed increment, and at 1, 5, 15, 30, and 60 min into the recovery. If blood collection was not feasible at a scheduled time point, that sample was omitted, and collection proceeded at the next designated time point. Plasma glucose and lactate were assayed for each time point, in real time, using a Pentra C400 Analyser (Horiba). HBA1c was assayed from whole blood, also in real time, on an Affinion2 (Abbott) the morning of each study.

### 2.4 Targeted metabolomics

Metabolites were extracted from plasma (10 µL) and concentrations obtained using the MxP 500 (Biocrates Life Science AG, Austria) following the manufacturer’s protocol. Metabolites were measured using a Nexera HPLC system (Shimadzu) coupled to a 6500+ QTRAP® mass spectrometer (AB Sciex) with an electrospray ionization source as previously described (Moaddel et al., 2022). Briefly, a 96-well based sample preparation device was used to quantitatively analyze the metabolite profile. Samples were analyzed by flow injection analysis-tandem mass spectrometry (FIA-MS/MS) and liquid chromatography-tandem mass spectrometry (LC-MS/MS) (Moaddel et al., 2022). Analytes in the LC-MS/MS part are quantified using either external 7-point calibration curves with labeled standards or internally with labeled standards (detailed information on the calibration is provided on the Biocrates website - www.biocrates.com). Analytes in the FIA-MS/MS part are quantified using internal standards. Concentrations were calculated using the Analyst/MetIDQ software and reported in µmol/L. Data were quantified using appropriate mass spectrometry software (Sciex Analyst®) and imported into Biocrates MetIDQ™ software for further analysis. The data was normalized to internal quality controls.

### 2.5 Statistical analysis

To evaluate temporal changes in metabolite levels, metabolites with more than 30% missing values were excluded as an initial filtering step to ensure data reliability. Mixed effects models were employed using the lme4 package in R, which accounts for repeated measurements within individuals. For each metabolite, two mixed effects models were fitted: a full model and a nested reduced model. Both models included subject as a random effect to account for individual differences, while “time,” “age,” and “sex,” which are consistent across individuals, were included as fixed effects. The reduced model excluded “time,” ensuring it was nested within the full model. All models were fitted using maximum likelihood estimation (ML) rather than restricted maximum likelihood estimation (REML) to facilitate model comparisons. To assess whether including specific factors, such as time or the interaction between time and fitness, significantly improved model fit, likelihood ratio tests (LRTs) were performed using the anova () function in R with the LRT option enabled. The resulting p-values indicated whether adding these factors significantly enhanced the model’s explanatory power. This approach was repeated for multiple comparisons, including differences between participants with high versus low fitness and testing the interaction between time and fitness. “Time” was modeled either as a categorical variable to capture non-linear effects or as a continuous variable when testing interactions with fitness level. Since LRTs do not indicate the direction of effects, separate mixed effects regression analyses were conducted for the low-fitness and high-fitness groups, with time (modeled as a continuous variable), age, and sex as fixed effects and subject as a random effect. Statistical significance was determined using the lmerTest package, which computes p-values based on Satterthwaite’s degrees of freedom method. For each metabolite, the beta coefficients for time were correlated between the high- and low-fitness groups, and the squared Pearson correlation coefficient (R^2^) was reported to quantify the relationship.

Participants were divided into high- and low-fitness groups based on the highest stage achieved during a fitness test. The high-fitness group consisted of three subjects who attained stage B6 or higher (two at B7 and one at B6) and was expanded to include nine subjects who reached B5, resulting in a total of 12 high-fitness subjects. The low-fitness group included seven subjects who reached B2, supplemented by ten subjects who achieved B3, yielding a total of 17 low-fitness subjects. Missing data for subjects who did not reach later stages (e.g., B3 for the low-fitness group or B6/B7 for the high-fitness group) were handled by the mixed effects modeling approach, which is robust to incomplete time points, thus obviating the need for imputation. All analyses, including regression and likelihood ratio tests, were conducted using these stratified groups.

We plotted all significant metabolites (40 from the peak exercise model and 38 from the recovery model) and visually tracked their dynamics, categorizing them into 4 patterns, regardless of whether the metabolites were significant in one or both models. Specifically, the results of the mixed effect models were group into four longitudinal patterns: “Difference maintained,” for metabolites that were already different between fitness groups and remained similarly different throughout all time points; “Baseline different,” for metabolites with similar blood levels at baseline that converged to similar level over time; “End different,” for metabolites that were similar at baseline but showed progressively increased difference with time; and “Switch,” when baseline levels were different between LF and HF and the expression pattern reversed with time.

Metabolite expression alterations were examined independently for LF and HF groups during two separate stages: the exercise period (roughly 9 min, from B0 to B3) and the recovery phase (1 h, from P1 to P60). The first 9 min of exercise was selected for this comparison as it allowed for the greatest number of participants to be represented. The HF group underwent an additional Exhaustion peak analysis, as these participants reached the 7th incline (B7). Visual representations of all five models, showcasing the top 10 upregulated and top 10 downregulated metabolites, can be found in (Supplementary Figure S1). A comprehensive list of all identified metabolites is provided in Supplementary Data S1, Sheet S1.

Microsoft Excel (Microsoft office) was used for data collection and Rstudio to analyze data, generate models and visualize plots.

## 3 Results

### 3.1 Metabolomic profiles in single-cross-sectional time point during early exercise and relative recovery

This investigation encompassed a cohort of 29 participants from the Baltimore Longitudinal Study of Aging (BLSA), with ages spanning 33–94 years. Participants were stratified into two fitness groups based on their time to exhaustion during the maximal exercise test (Figure 1B; Supplementary Table S1), with Low Fitness (LF) participants (n = 17) reaching exhaustion within 9 min (B3) and High Fitness (HF) participants (n = 12) able to maintain sustained exercise for at least 15 min.

Participants in LF were older, had lower VO_2_ peak and higher HbA1c and percentage body fat compared to HF participants. No significant differences were observed for height, weight and BMI (Figure 1C). Overall study design is depicted in Figure 1A. As expected, lactic acid exhibited a robust increase at the onset of exercise with a full recovery following exercise in both groups; however, maximum lactate levels were much increased at the end of the maximum exercise period for the HF group (Figure 1D). Glucose increased after B3 and decreased during recovery in the HF group (Figure 1E).

### 3.2 Fitness-stratified metabolic signatures reveal enhanced early exercise metabolite circulation in low fitness individuals despite recovery overlap

**FIGURE 2:**
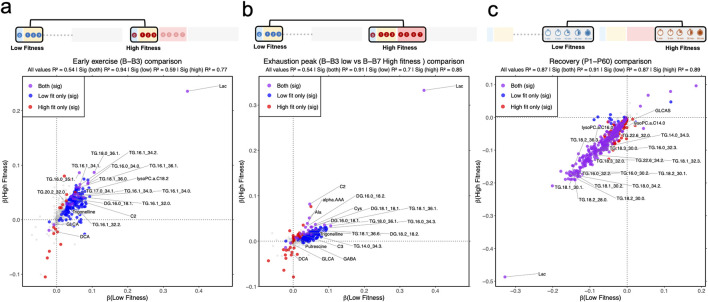
Correlation between trend of significant metabolic dynamics in low- and high-fitness groups during early exercise and recovery. **(a)** Linear model in low- and high-fitness, separately, for baseline, B1, B2, and B3. **(b)** Linear model in low- and high-fitness, separately, for baseline, P1, P5, P15, P30, and P60. **(c)** Linear model in low- and high-fitness, separately, for 1, 5, 15, 30 and 60 minutes recovery (p1, p5, p15, p30 and p60). Metabolite classifications: gray (non-significant in both groups), red (high-fitness-specific significance), blue (low-fitness-specific significance), purple (significant in both groups).

The beta coefficients estimating changes of metabolites from baseline to the stage of B3 were significantly different from zero for 134 unique metabolites, the LF group and 16 unique metabolites in the HF group, with 22 metabolites common to both groups (Figure 2A; Supplementary Data S1). When we compared each group at their respective peak exhaustion points—stage B3 for the LF group and stage B7 for the HF group—rather than limiting both groups to the first 3 stages, we still found minimal overlap (Figure 2B). Only 21 metabolites showed significant changes in both groups, while 29 metabolites changed significantly only in the HF group. Notably, 7 bile acids showed decreased levels (downregulation) in the High Fitness (HF) group, both when measured up to stage B3 and when measured up to their exhaustion point (B7). Of 7 bile acids, only 2—glycolithocholic acid (GLCA) and deoxycholic acid (DCA)—also showed significant changes in the Low Fitness (LF) group at their exhaustion point. Beyond GLCA and DCA, only 5 other metabolites showed significant changes across all 3 comparison groups (LF from baseline to B3, HF from baseline to B3, and HF from baseline to B7). These metabolites were: lactic acid, acetyl carnitine, diglyceride 16:0_18:1 (DG 16:0_18:1), triglyceride 18:0_36:1 (TG 18:0_36:1), and trigonelline. All of these metabolites changed in the same direction across all groups (Supplementary Data S1, Sheet S3).

During recovery, the metabolomic profiles of both fitness groups converged substantially, with 322 metabolites showing changes in both LF and HF groups (Figure 2C). We found that 14 metabolites changed exclusively in the LF group, and 75 metabolites changed exclusively in the HF group. For example, when looking at the classes of metabolites, several were following the same pattern, where they were decreasing in both fitness groups: ceramides (11 in HF, 8 in LF); HexCer (13 in HF, 6 in LF); DGs (6 in HF, 5 in LF); LPCs (10 in HF, 9 in LF); PCs (64 in HF, 56 in LF); SMs (14 in HF, 9 in LF); TGs (219 HF, 199 LF) and lactic acid, of which only HexCer d16:1/22:0 and TG 20:4/33:2 were the only metabolites to be specifically identified in the LF group. The BCAAs were also decreasing in both fitness groups, with only valine not significant (p = 0.10) in the LF group.

### 3.3 Linear mixed model interaction analysis of low and high fitness identifies distinct metabolic dynamics

**FIGURE 3:**
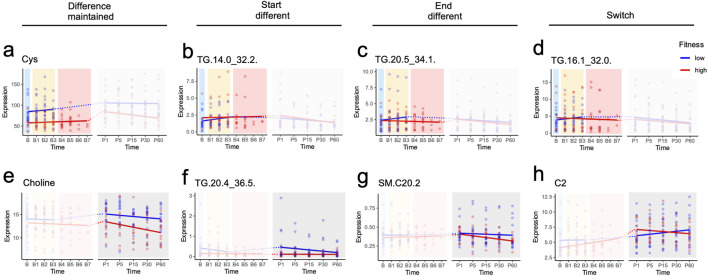
Metabolic responses across study design phases identified by mixed linear model analysis. **(a-h)** show metabolites that reached statistical significance in at least one of three analytical models: early exercise (B-B3), peak exercise (B-B2/B3 for low fitness; B-B5/B6/B7 for high fitness), and recovery (1–60 min post-exercise). Dotted lines connect the final exercise timepoint to the first recovery timepoint; these connections span different models and were not included in statistical analyses. Significance levels: p ≤ 1, *p ≤ 0.05, **p ≤ 0.01, ***p ≤ 0.001.

Further analyses were done to investigate metabolites that differentially changed over time in the LF and the HF groups though mixed effect models. Two distinct models were implemented. The first approach explored the effect of exercise from baseline to exhaustion (Supplementary Figure S2) and the second the effect of recovery between the LF and HF groups (Supplementary Figure S3). Overall, 40 metabolites showed significant differential changes in the peak exercise model and 38 metabolites in the recovery model. Figure 3 illustrates the four categories, presenting one significant (p < 0.05) example for each category from either the Exercise or Recovery model. After establishing this categorization, we generated a Summary Table to interpret the results (Table 1).

**TABLE 1:** Low vs. High fit interaction model.

Metabolite category	Difference maintained		Start different		End different		Switch	
	Exercise	Recovery	Exercise	Recovery	Exercise	Recovery	Exercise	Recovery
Triacylglycerols	-	-	TG18:0_30:1*	TG18:0_30:1*	-	TG16:0_40:7	TG16:1_32:0	-
	-	-	TG18:1_26:0*	TG18:1_26:0*	TG16:0_32:3	-	TG20:5_36:3	-
	-	-	TG18:1_30:2*	TG18:1_30:2*	TG16:0_33:2	-	TG22:5_34:1	-
	-	-	TG14:0_32:2	-	TG16:1_32:2	-	-	-
	-	-	TG14:0_34:2	-	TG17:0_34:2	-	-	-
	-	-	TG14:0_36:1	-	TG17:1_34:3	-	-	-
	-	-	TG16:0_28:1	-	TG17:1_36:3	-	-	-
	-	-	TG16:0_28:2	-	TG17:1_36:4	-	-	-
	-	-	TG16:0_30:2	-	TG18:0_34:3	-	-	-
	-	-	TG16:0_32:2	-	TG18:3_32:1	-	-	-
	-	-	TG18:0_32:1	-	TG20:1_34:3	-	-	-
	-	-	TG18:0_32:2	-	TG20:5_34:1	-	-	-
	-	-	TG18:1_28:1	-	TG20:5_34:2	-	-	-
	-	-	TG18:1_30:1	-	-	-	-	-
	-	-	TG18:2_28:0	-	-	-	-	-
	-	-	TG18:2_30:0	-	-	-	-	-
	-	-	TG18:2_30:1	-	-	-	-	-
	-	-	-	TG20:0_34:1	-	-	-	-
	-	-	-	TG20:4_36:3	-	-	-	-
	-	-	-	TG20:4_36:5	-	-	-	-
	-	-	-	TG20:5_36:3	-	-	-	-
	-	-	-	TG18:0_38:6	-	-	-	-
Bile acids	GCA	-	TCA	TCDCA	-	-	-	-
	-	TCA	-	TDCA	-	-	-	-
	-	-	-	TLCA	-	-	-	-
	-	-	-	GCA	-	-	-	-
	-	-	-	GCDCA	-	-	-	-
	-	-	-	GDCA	-	-	-	-
	-	-	-	GLCA	-	-	-	-
Ceramides, sphingomyelins	-	-	-	SM-OH C14:1	-	SM C20:2	Cer d18:0_24:1	-
	-	-	-	SM-OH C22:1	-	-	-	Cer d18:1_26:0
	-	-	-	SM-OH C22:2	-	-	-	Hex3_Cer d18:1_16:0
	-	-	-	SM-OH C24:1	-	-	-	-
Aa-related metabolites, biogenic amines	Cys*	Cys*	-	a-aaa	-	-	-	Trigonelline
	-	Choline	-	-	-	-	-	Trp
Phospoholypids	-	PCaaC36:5	-	Pcae C34:3	-	-	-	-
	-	PCaeC36:5	-	PCaeC36:4	-	-	-	-
Carnitines	-	-	-		-	C0	-	C2
	-	-	-		-	-	-	C3
Carboxilic and fatty acid	-	-	-	Lac	-	-	DHA	-
Cholesterol esters	-	-	-	CE 15:0	-	-	-	-

Of interest, only 4 metabolites had the same classification in exercise and recovery. Cysteine was the only metabolite that maintained differences across both fitness groups during exercise and recovery, while TGs (TG 18:0_30:1, TG 18:1_26:0 and TG 18:1_30:2) started different at the onset of both exercise and recovery and ended not significantly different (Table 1). While it may seem surprising that these TGs at B3 were similar but started different at P1, this may be due to the response to exhaustion in the LF group (which ends at B3) versus the HF group (which can go to B7).

Interestingly, in both exercise and recovery, the metabolomic changes predominantly observed were in TGs, bile acids and SMs. Only 17 TGs had baseline line levels that were different, with 14 TGs similar at the end of exercise and throughout recovery and 3 switching at the end of exercise. There were 5 TGs that were similar at baseline, but were different at the start of the recovery, except for TG 20:5_36:3. Of the bile acids, only TCA and GCA had different profiles, with GCA maintaining its baseline difference throughout exercise while they converged to be similar between LH and HF at the end of recovery. TCA, on the other hand, started different at baseline during exercise but converged to similar levels between the two group at the end (B3 for LF and B7 for HF). Similar to the TGs discussed above, the difference in the level of exhaustion (B3 for LF and B7 for HF), may explain why they may have been similar by B3 but resulted in different baseline levels at P1. The rest of the bile acids were similar at baseline; however, circulating levels were different at the start of recovery but by the end of recovery both groups had similar levels. Of the ceramides and SMs, at baseline only Cer d18:0_24:1 was different between the LF and HF groups. At the start of recovery 4 hydroxy-SMs, Cer d18:1_26 and Hex3_Cerd18:1_16:0 were different, whereas SM 20:2, and three ceramides were different at the end of recovery.

Overall, our findings suggest that the classes of metabolites that changed differentially between LF and HF included predominantly, TGs, and bile acids, and a few ceramides and SMs.

## 4 Discussion

In this study, we identified metabolomic changes, predominantly consisting of TGs, bile acids and SMs and ceramides, that were either shared or specific to high fitness (HF) versus low fitness (LF) groups in healthy middle-aged and older adults.

One striking physiological difference captured by this investigation is the exercise-associated changes in circulating TG levels. During exercise, TGs are released from adipose tissue stores and enter the bloodstream. They are then translocated across the muscle cell membrane by fatty acid translocases to support intracellular ATP generation (Egan et al., 2013; Romijn et al., 1993; Romijn et al., 1995). Thus, changes in the circulating concentration of TGs during exercise are the result of changes in the rate of their liberation from adipose tissue (i.e., lipolysis) and uptake by skeletal muscle tissue. In our study, the differences in the TGs followed different profiles in that participants with higher levels of physical fitness maintained steady state circulating TG levels throughout increasing exercise intensities, indicating an exquisite balance between the rates of lipolysis and uptake by skeletal muscle. In the individuals with lower fitness (Figure 2), however, circulating TG levels increased at the onset and throughout exercise. It is likely that in the skeletal muscle of the LF individuals, there was an increased reliance on intramuscular glycogen and TG stores due to the elevated relative intensity of exercise at lower absolute workloads (Egan et al., 2013; Romijn et al., 1993; Romijn et al., 1995). Linear regression analysis in the fitness groups indicates that the magnitude of metabolic changes to the onset of exercise is related to fitness levels (Supplementary Figure S2). Moreover, the LF group may have increased lipoprotein lipase activity due to a degree of insulin resistance in skeletal muscle, thereby limiting fatty acid uptake into this tissue (Kim et al., 2001). Participants in LF as expected had larger metabolomic changes from baseline to B3, consistent with the LF group exercising near or at their VO_2_max, resulting in differences in metabolic responsiveness between LF and HF at 9 min (exhaustion for LF).

Another notable finding from this investigation is that the more fit adults had lower circulating levels of both bile and certain amino acids at baseline (Supplementary Figure S2), and they remained lower through increasing exercise intensities as well as at 1 hour of recovery compared to LF group. This pattern aligns with previous research showing lower circulating bile acid levels in physically active adults compared to less active individuals (Danese et al., 2017). It is difficult to speculate on underlying mechanisms, but possible mechanisms may include a change in gut microbiota composition, which has been shown to play a role in affecting cardiorespiratory fitness in healthy young adults (Durk et al., 2019) or perhaps a blood flow redistribution between different organs during exercise (Joyner and Casey, 2015). The literature suggests that these findings are most likely driven by the relative redistribution of tissue blood flow during exercise away from the splanchnic bed, which would reduce the net appearance of bile acids into the bloodstream (Joyner and Casey, 2015; Rowell et al., 1965; Rowell et al., 1984). In addition, there were notable differences as well including the change in CEs, where 10 were decreasing in the HF group and none in the LF group, consistent with exercise increasing cholesterol uptake by hepatocytes in HF group. Acylcarnitines (C0, C2, C3, C4) were also decreasing in HF group, with only C2 significantly changing (increasing) in the LF group. The continued increase of C2 during recovery in the LF suggests stronger exercise-induced homeostatic perturbations. Several studies have shown an association with circulating levels of ceramides, sphingomyelins and exercise (Bergman et al., 2015; Carrard et al., 2021; Reidy et al., 2020). While there were relatively few sphingomyelins/ceramides that were different between HF and LF group, exercise has been shown to lead to a reduction of ceramides in circulation (Carrard et al., 2021). Also, in our study, most changes were observed during recovery, consistent with results from a publication that reports decreased levels during recovery (Bergman et al., 2015).

This study has limitations. Age-matched and fitness-matched cohorts could potentially shed further insight on the influence of these components on the circulating metabolome. The influence of blood flow redistribution between tissues is an important confounding variable that is difficult to adequately correct for without invasive measures of real-time tissue-specific blood flow. For these reasons, we cannot definitively conclude that any individual metabolite or metabolic pathway is a primary driver of age-associated fatigability. However, this investigation provides a comprehensive time course of changes in the circulating metabolome in a group of well-characterized, healthy older adults.

In summary, this investigation provides novel temporal profiles of the metabolomic response to increasing exercise intensities to volitional exhaustion, and throughout recovery from exercise in healthy middle-aged and older individuals. The current data indicate that older adults of varying fitness levels have distinct metabolomic responses to the onset of aerobic exercise and through gradually increasing exercise intensities. These distinctions are especially prevalent in the trajectory of bile acids and lipids (Figure 2). These data are potentially useful in laying the foundation for development of biomarkers for physical fitness that can be detected in the early stages of an exercise test. To continue the development of clinically useful screening panels for physical fitness in older adults it is important to further refine this approach, continually implement metabolomic profiling, and correlate unique patterns of metabolic responses with the development of future comorbidities and mortality.

## Data Availability

The raw data supporting the conclusions of this article will be made available by the authors, without undue reservation.

## Supplementary material

SUPPLEMENTARY FIGURE S1
Metabolic dynamics during exercise and recovery in low- and high-fitness groups. **(a–b)** Volcano plots comparing differential metabolite expression (log_2_ fold change vs. −log_10_ p-value) for **(a)** low-fitness and **(b)** high-fitness groups during early exercise. **(d–e)** Volcano plots for **(d)** low-fitness and **(e)** high-fitness groups during recovery (1, 5, 15, 30, and 60 minutes post-exhaustion). Significantly upregulated metabolites (adjusted p < 0.05) appear in red (right side of quadrants), while downregulated metabolites are in blue (left side of quadrants). Dashed lines represent p = 0.05 thresholds.

SUPPLEMENTARY FIGURE S2
Plot of metabolic responses during baseline to peak of exhaustion (exercise phases, B-B2/B3 for low fitness; B-B5/B6/B7 for high fitness). Only significant (p<0.05) metabolites are visualized.

SUPPLEMENTARY FIGURE S3
Plot of metabolic responses during recovery phase (up to 1 hour post peak of exhaustion during exercise). Only significant (p<0.05) metabolites are visualized.
