# Supplementary material for: Plasma longitudinal metabolic changes with acute maximal aerobic exercise and one-hour recovery
Source: Front Mol Biosci. 2025 Jul 11;12:1613238. doi: 10.3389/fmolb.2025.1613238 (PMC12289478; doi:10.3389/fmolb.2025.1613238)
Supplement: Supplementary file 1 [file Table1.docx]

| ID | Age (years) | Fitness cohorts | Sex | Race |
| --- | --- | --- | --- | --- |
| 1 | 30 | High | Male | Caucasian |
| 2 | 41 | Low | Female | Caucasian |
| 3 | 47 | High | Female | Caucasian |
| 4 | 49 | High | Male | Caucasian |
| 5 | 49 | High | Female | Caucasian |
| 6 | 51 | High | Female | Caucasian |
| 7 | 53 | High | Male | African American |
| 8 | 58 | High | Male | Asian |
| 9 | 63 | High | Female | Caucasian |
| 10 | 64 | High | Female | Caucasian |
| 11 | 67 | Low | Male | Caucasian |
| 12 | 69 | High | Male | Caucasian |
| 13 | 75 | High | Male | Caucasian |
| 14 | 75 | Low | Female | Caucasian |
| 15 | 75 | High | Female | Caucasian |
| 16 | 77 | Low | Female | Caucasian |
| 17 | 77 | Low | Male | African American |
| 18 | 80 | Low | Female | Caucasian |
| 19 | 81 | Low | Female | Caucasian |
| 20 | 81 | Low | Male | Caucasian |
| 21 | 82 | Low | Male | African American |
| 22 | 82 | Low | Male | Caucasian |
| 23 | 82 | Low | Male | Caucasian |
| 24 | 85 | Low | Male | Caucasian |
| 25 | 85 | Low | Male | Caucasian |
| 26 | 85 | Low | Female | Caucasian |
| 27 | 85 | Low | Male | Caucasian |
| 28 | 88 | Low | Male | Caucasian |
| 29 | 94 | Low | Female | Caucasian |

**Supplementary Table 1.** Population study demographics
