# Supplementary figures and images for: Plasma longitudinal metabolic changes with acute maximal aerobic exercise and one-hour recovery
Source: Front Mol Biosci. 2025 Jul 11;12:1613238. doi: 10.3389/fmolb.2025.1613238 (PMC12289478; doi:10.3389/fmolb.2025.1613238)

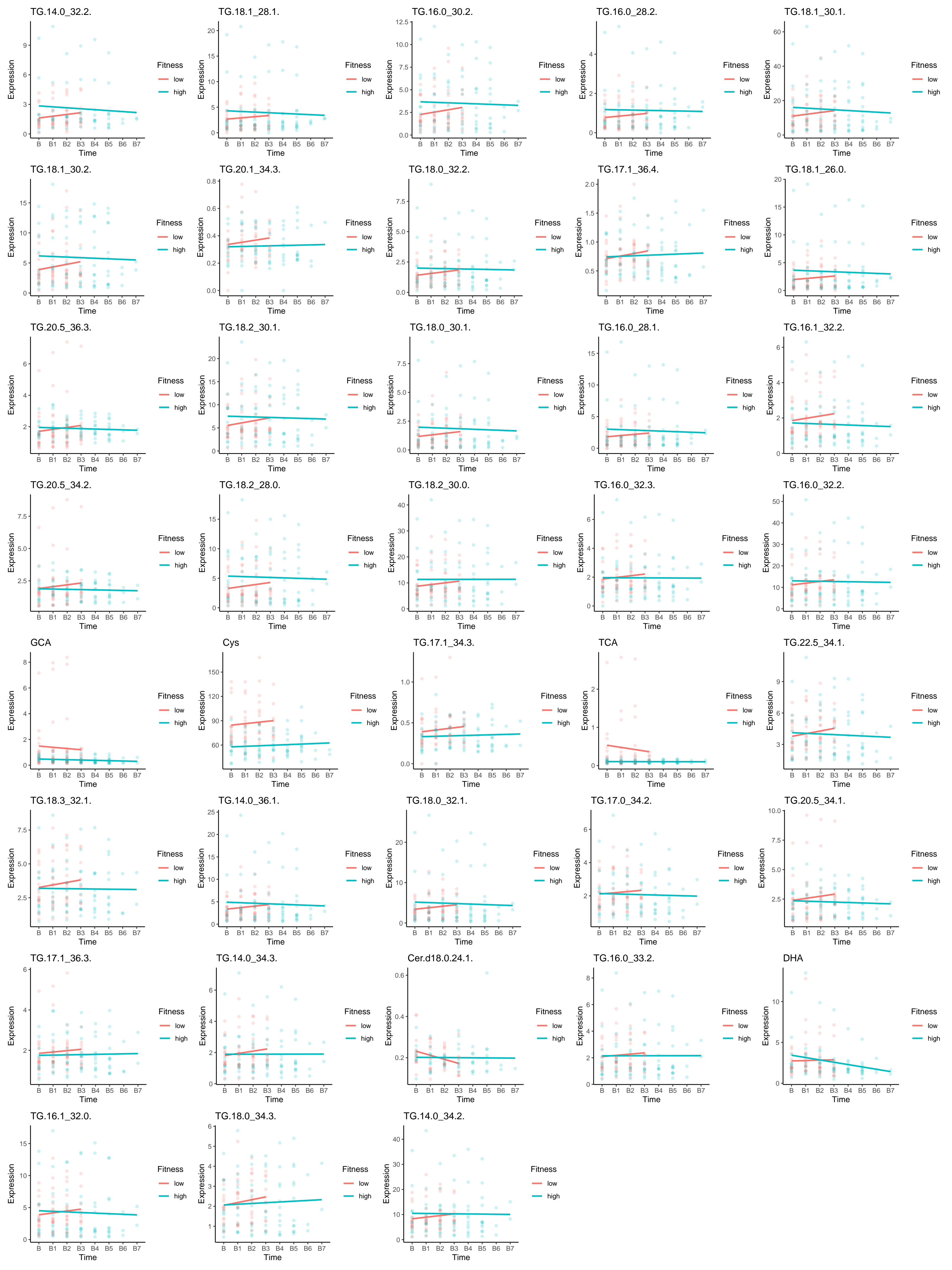

Supplement: Supplementary file 2 [file Image2.pdf]

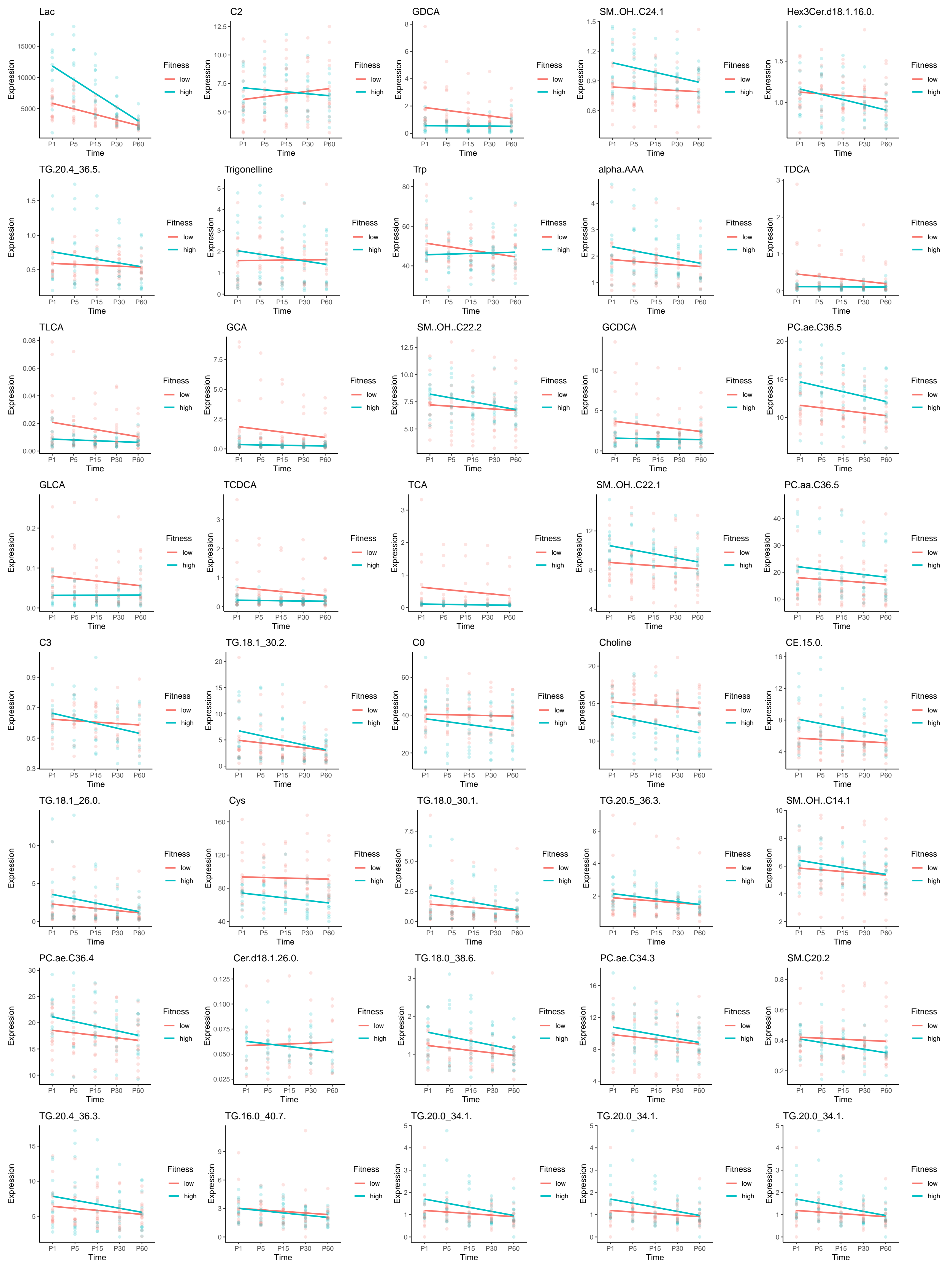

Supplement: Supplementary file 3 [file Image3.pdf]

Low fitness

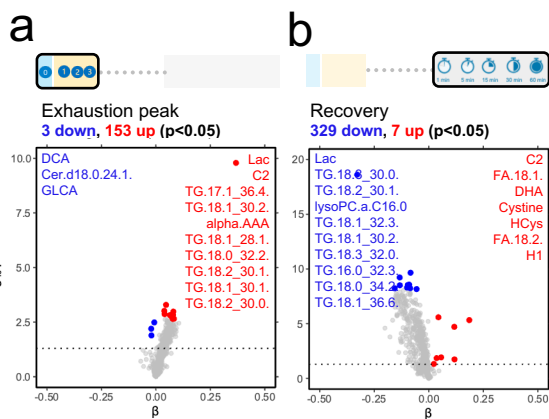

High fitness

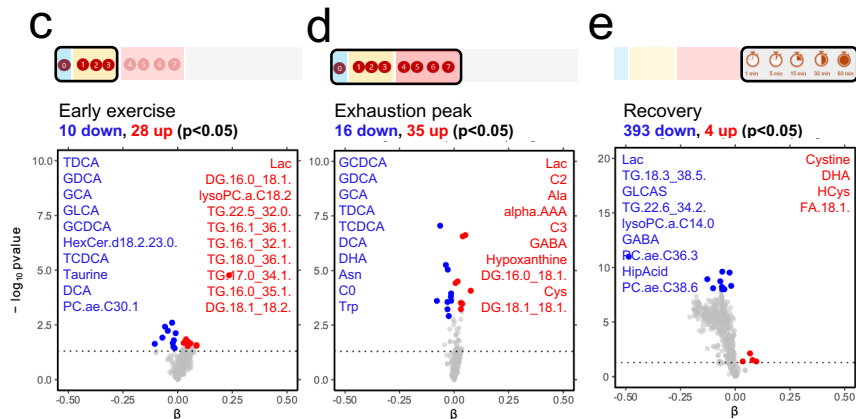

Supplement: Supplementary file 4 [file Image1.pdf]
